# Nanocalorimetric Characterization of Microbial Activity in Deep Subsurface Oceanic Crustal Fluids

**DOI:** 10.3389/fmicb.2016.00454

**Published:** 2016-04-05

**Authors:** Alberto Robador, Douglas E. LaRowe, Sean P. Jungbluth, Huei-Ting Lin, Michael S. Rappé, Kenneth H. Nealson, Jan P. Amend

**Affiliations:** ^1^Center for Dark Energy Biosphere Investigations, NASA Astrobiology Institute, University of Southern California, Los AngelesCA, USA; ^2^Department of Earth Sciences, University of Southern California, Los AngelesCA, USA; ^3^Department of Oceanography, School of Ocean and Earth Science and Technology, University of Hawaii at Manoa, HonoluluHI, USA; ^4^Hawaii Institute of Marine Biology, School of Ocean and Earth Science and Technology, University of Hawaii at Manoa, KaneoheHI, USA; ^5^Department of Biological Sciences, University of Southern California, Los AngelesCA, USA

**Keywords:** calorimetry, crustal fluids, microbial energy turnover, subsurface biosphere, cell-specific metabolic rates

## Abstract

Although fluids within the upper oceanic basaltic crust harbor a substantial fraction of the total prokaryotic cells on Earth, the energy needs of this microbial population are unknown. In this study, a nanocalorimeter (sensitivity down to 1.2 nW ml^-1^) was used to measure the enthalpy of microbially catalyzed reactions as a function of temperature in samples from two distinct crustal fluid aquifers. Microorganisms in unamended, warm (63°C) and geochemically altered anoxic fluids taken from 292 meters sub-basement (msb) near the Juan de Fuca Ridge produced 267.3 mJ of heat over the course of 97 h during a step-wise isothermal scan from 35.5 to 85.0°C. Most of this heat signal likely stems from the germination of thermophilic endospores (6.66 × 10^4^ cells ml^-1^_FLUID_) and their subsequent metabolic activity at temperatures greater than 50°C. The average cellular energy consumption (5.68 pW cell^-1^) reveals the high metabolic potential of a dormant community transported by fluids circulating through the ocean crust. By contrast, samples taken from 293 msb from cooler (3.8°C), relatively unaltered oxic fluids, produced 12.8 mJ of heat over the course of 14 h as temperature ramped from 34.8 to 43.0°C. Corresponding cell-specific energy turnover rates (0.18 pW cell^-1^) were converted to oxygen uptake rates of 24.5 nmol O_2_ ml^-1^_FLUID_ d^-1^, validating previous model predictions of microbial activity in this environment. Given that the investigated fluids are characteristic of expansive areas of the upper oceanic crust, the measured metabolic heat rates can be used to constrain boundaries of habitability and microbial activity in the oceanic crust.

## Introduction

Low to moderate temperature (<100°C) fluids circulating within the uppermost basaltic ocean crust are known for their contribution to global biogeochemical cycling (Bach and Edwards, 2003; Orcutt et al., 2011; Robador et al., 2015), and for harboring an expansive microbial biosphere distinct from marine sediments (Jungbluth et al., 2016). Knowledge of the metabolic reactions supporting life in these habitats, however, is sparse and limited to specific respiration processes such as oxygen consumption, methane cycling, and sulfate reduction (Lever et al., 2013; Orcutt et al., 2013b; Robador et al., 2015). A better understanding of the metabolic activity in deep subseafloor fluids is handicapped by several technical challenges, including access to these habitats and the suspected slow growth rates of microorganisms in such low-energy environments (Cowen, 2004).

The physiological state of microbial communities in low-energy systems is manifestly different than those observed in laboratory cultures, which are characterized by rapid growth, high metabolic rates, and high cell densities (Neidhardt et al., 1990). Natural, energy-starved microbial communities are typically associated with a minimal array of functions required to sustain a metabolically active state, or basal maintenance power (Hoehler and Jørgensen, 2013). As a result, it becomes difficult to determine whether microorganisms in energy-limiting environments are alive and active (Jørgensen, 2011). Therefore, one corresponding research challenge is the development of technical approaches wherein microbial metabolism can be characterized. Current techniques for the quantification of microbial activities rely on the identification of specific turnover processes that often occur simultaneously (e.g., methanogenesis and sulfate reduction, Lovley et al., 1982) at rates below detection limits (Adhikari and Kallmeyer, 2010).

An alternative approach to determining the metabolic rate of microorganisms in low-energy settings is to measure the energy dissipated by all biological activities using direct calorimetry (Brown et al., 2004). This approach has been used to accurately measure the heat generated from metabolic activity in microbial cultures (Winkelmann et al., 2004; Braissant et al., 2010) but, despite recent advances in quantifying microbial respiration rates in the environment (Djamali et al., 2012; Mukhanov et al., 2012), technical limitations such as low sensitivities and slow responses have limited the application of calorimetry for these measurements (Karl, 2014).

Calorimetry is based on the assumption that heat is the direct product of all metabolic functions (Russell, 1986; Russell and Cook, 1995). Calorimetry data provide direct heat measurements, which are indicative of the change in enthalpy associated with microbial activity, therefore, allowing for a direct quantification of microbial energy turnover. So far, microbial energy consumption in low-energy environments has been estimated from the turnover rates of an electron donor or acceptor and the calculated Gibbs free energy yield of the predicted reaction (Orcutt et al., 2013a; Lin et al., 2014; LaRowe and Amend, 2015a,b). Calorimetry measurements, however, are not confounded by certain problems of Gibbs free energy calculations (Russell and Cook, 1995) such as those resulting from uncertainties in the exact stoichiometry of the reaction, in the concentrations of reactants and products, and in the estimation of activity coefficients and thermodynamic properties for all individual chemical species.

**FIGURE 1:**
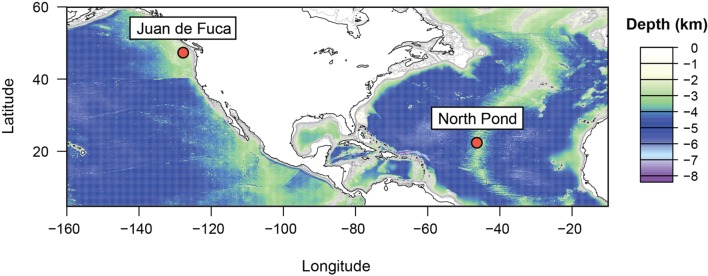
**Location of CORK observatory sampling sites on the Juan de Fuca Ridge flank, Pacific Ocean (47°45.6′N, 127°45.6′W) and the North Pond, Atlantic Ocean (22°48.1′N, 46°03.2′W)**.

In this study, we measured the change in enthalpy as a function of temperature by direct nanocalorimetry in order to quantify and interpret microbial energy requirements in natural subseafloor igneous crustal fluids from two aquifers that differ with respect to their *in situ* physicochemical conditions. Warm (>60°C) anoxic fluids are common in all ocean basins (Davis et al., 1997); however, the majority of the upper oceanic crust experiences much cooler (<20°C, Johnson and Pruis, 2003) and presumably oxic conditions (Ziebis et al., 2012; Orcutt et al., 2013b). Therefore, we examined the metabolic heat production rates of microorganisms in highly geochemically altered fluids that are characteristic of the Juan de Fuca Ridge (JFR) in the eastern Pacific Ocean, but focused on fresh-to-moderately altered fluids from a site known as North Pond (NP) in the northern Atlantic Ocean (**Figure 1**), which may be more informative ecologically to constrain the global rates of microbial activity in deep basaltic ocean crust.

## Materials and Methods

### Sampling

Crustal fluids were collected from subseafloor Circulation Obviation Retrofit Kit (CORK) observatories installed within boreholes drilled several hundred meters into the ocean crust, preventing circulation between the open hole and ocean bottom water and allowing access to deep crustal fluids. Samples from the eastern flank of the JFR were obtained in August 2014 from a CORK observatory at borehole U1362A (47°45.6′N, 127°45.6′W) using the ROV *Jason II* deployed from the R/V *Atlantis* (cruise AT 26-18). NP crustal fluids were sampled in April of 2012 from a CORK observatory at Hole U1383C (22°48.1241′N, 46°03.1662′W) using the ROV *Jason II* (WHOI) deployed from the R/V *Maria S. Merian* (cruise MSM 20-5). Both boreholes (U1362A and U1383C) are instrumented with multilevel CORK observatories (Fisher et al., 2011; Edwards et al., 2014), which allow the sampling at different depth intervals using umbilicals with non-reactive tubing (Teflon^®^ and Tefzel^®^, DuPont^TM^, for U1362A and U1383C respectively). The CORK observatory at borehole U1362A (JFR) is situated at 2661 m below sea level, penetrating 236 m of sediment and the upper 292 m of basement. The CORK observatory at borehole U1383C (NP) is located at 4425 m below sea level and penetrates the upper 293 m of basement through 38 m of sediment. Samples from the deepest horizons were collected at the seafloor into sampling bags of Tedlar polyvinyl fluoride (PVF) film (Midan Co., Chino, CA, USA) as previously described (Robador et al., 2015) and returned to the ship using an independent elevator. Whole crustal fluids were subsequently transferred by gravity feed to 2 L glass bottles (previously cleaned and combusted at 480°C for 6 h) sealed with butyl rubber stoppers and stored at 4°C until further processing.

### Crustal Fluids

Crustal fluids circulating through the eastern flank of the JFR system are warm (63°C) and characterized by a steep chemical gradient; the dominant oxidant changes from oxygen to sulfate, which is accompanied by a decrease in organic matter concentration and an increase in the reduced species hydrogen sulfide and methane (Robador et al., 2015). Crustal fluids within upper basaltic basement at NP on the other hand are younger, much cooler (3.8°C), and oxic (Meyer et al., 2016). Fluids collected from CORK observatories at boreholes U1362A and U1383C were analyzed for the major and minor chemical constituents in seawater. Analytical data used in this study are compiled in **Table 1**. Additional original data, including analytical methods, are published elsewhere (Edwards et al., 2014; Lin et al., 2014; Meyer et al., 2016).

**Table 1:** Chemical compositions of basaltic crustal fluids collected from CORK observatories at boreholes U1362A and U1383C.

	U1362A^a^	U1383C^b^
Temperature (°C)	63	3.8
Pressure (kpa)	∼27,071	∼46,815
pH	7.9	7.6
**Dissolved constituents**		
Ca^2+^ (mM)	55.1	10.1
SO_4_^2-^ (mM)	18.0	27.6
Na^+^ (mM)	463.2	459
K^+^ (mM)	6.4	10
Mg^2+^ (mM)	2.2	52.4
Total organic carbon (μM)	15.5	24.2
Total nitrogen (μM)	112.0	24
Si (μM)	1176.0	120
NO^3-^ (μM)	<0.1	21.8
O_2_ (μM)	<0.5	213


*^a^This study.*

*^b^Data from Edwards et al. (2014) and Meyer et al. (2016).*

### Nanocalorimetry Experiments

Metabolic heat production rates in crustal fluids were measured using a thermal activity monitor model TAM III equipped with a nanocalorimeter (TA Instruments, Lindon, UT, USA). The TAM III coupled to the nanocalorimeter offers the maximum sensitivity of most commercial isothermal calorimeters (Braissant et al., 2010). These metabolic heat production rates are measured in a pair of sealed glass ampules (up to 4 ml) as the difference in heat flow between the sample and a reference.

Two-step isothermal experiments were performed on the same sample at different temperatures (15–100°C for JFR and 30–85°C for NP). These temperature ranges extend beyond the upper and lower limits of observed microbial activity. To ensure that the samples were in thermal equilibrium with the calorimeter and that the collected data corresponded to that at the true transition temperatures, a very slow scan rate (0.5°C h^-1^) was used. Thermal equilibrium is not always achieved when differential scanning calorimetry data are collected using far higher scanning rates (often 15–60°C h^-1^, Hansen and Criddle, 1990; Mukhanov et al., 2012).

Calorimetry experiments consisted of 2 ml of unamended crustal fluids and 2 ml of headspace in glass ampules (previously cleaned and combusted at 480°C for 6 h), crimped and sealed with butyl rubber stoppers. To prevent contamination of the JFR flank anoxic fluids with oxygen, the ampules were prepared in an anaerobic chamber (<5 ppm oxygen and 5% hydrogen gas mix, COY Laboratory Products Inc., Grass Lake, MI, USA). The reference ampule consisted of 2 ml filter-sterilized (0.2 μm) crustal fluids and 2 ml of headspace. Sample and reference ampules were prepared at the same time (i.e., a new reference was used for each measurement) and pre-incubated in the calorimeter at the operating temperature for 24 h prior to each experiment. This minimized potential changes in heat flow associated with, for example, differences in temperature between sample and nanocalorimeter and stresses related to sample preparation.

Two independent nanocalorimeters were used simultaneously to run parallel blank experiments, which consisted of 0.2 μm filter-sterilized crustal fluids in both the sample and reference ampules. An initial baseline correction was done by subtracting the blanks from the sample heat-flow profiles. After identification of upper and lower temperatures of measurable metabolic heat rates, a second baseline correction was drawn manually between these temperatures using the TAM III Lab Assistant Software (V1.3.0.153, TA Instruments, Lindon, UT, USA) to obtain final data on microbial activity.

### Quantifying Spore-Forming Bacteria

Endosporulation allows certain bacteria to persist as dormant cells in adverse conditions, such as prolonged exposure to the low (4°C) storage temperatures (Hubert et al., 2009). In order to quantify the numerical contribution of spore-forming bacteria in our experiments, 150 ml of JFR and NP fluids were pasteurized at 80°C for 2 h and incubated at 50°C for 48 h to induce germination of spores as previously described (Müller et al., 2014). Germinated cells were subsequently quantified as described below.

### Total Cell Counts

Cell counts, in triplicate, were performed as previously described for marine planktonic environments (Patel et al., 2007). In short, 10–150 ml of crustal fluids were sampled and fixed in 0.2 μm filtered formalin [37–39% (wt/vol) formaldehyde solution] overnight at 4°C. Samples were then filtered onto polycarbonate membrane filters (type, GTBP; pore size, 0.2 μm; diameter, 2.5 mm; Sartorius, Göttingen, Germany) and stained with SYBR Green I (1:400 dilution from stock solution; Life Technologies, Carlsbad, CA, USA). Filters were mounted onto microscope glass slides with 0.1% (vol/vol) *p*-phenylenediamine anti-fade mounting medium and visualized using a Nikon Eclipse Ti-E inverted microscope (Nikon, Tokyo, Japan) equipped with a drift correction unit (Nikon Perfect Focus System) for maintaining focus at the coverslip-filter interface during imaging. Fluorescence imaging of SYBR Green I was done in the FITC (Nikon filter set B-2E/C). At least 1000 cells stained with SYBR Green I were counted per sample using the scientific image analysis and visualization program *DAIME* (Daims et al., 2006).

### Thermodynamic Calculations

**FIGURE 2:**
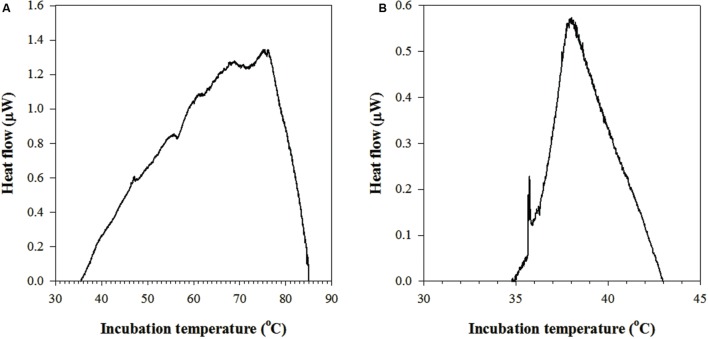
**Metabolic heat rates as a function of temperature as measured by two-step isothermal nanocalorimetry in subseafloor crustal fluids of **(A)** Juan de Fuca Ridge flank and **(B)** North Pond.** An initial baseline correction was performed by subtracting blanks from sample heat-flow profiles. After identification of upper and lower temperatures of measurable metabolic heat rates, a second baseline correction was performed manually between these temperatures.

Enthalpies (Δ*H*_r_) of organic matter oxidation with the dominant available terminal electron acceptor (SO_4_^2-^ for JFR and O_2_ for NP) were calculated as a function of temperature for the reactions

$$4C_{5}H_{9}{NO}_{2}+{11SO}_{4}^{2-}+{8H}_{2}O\to 11{HS}^{-}+20{HCO}_{3}^{-}+5H^{+}+4{NH}_{4}^{+}$$

$$2C_{5}H_{9}{NO}_{2}+11O_{2}+{4H}_{2}O\to 10{HCO}_{3}^{-}+8H^{+}+2{NH}_{4}^{+}$$

$$2C_{5}H_{9}{NO}_{2}+15O_{2}+2H_{2}O\to 10{HCO}_{3}^{-}+12H^{+}+2{NO}_{3}^{-}$$

respectively (see **Figure 2**). Proline (C_5_H_9_NO_2_) was used as a proxy for organic matter since its nominal carbon oxidation state (-0.4, see LaRowe and Van Cappellen, 2011) is the same as that of the dissolved organic carbon in JFR fluids (personal communication, Boris Koch). Values of Δ*H*_r_ were calculated as a function of temperature using the revised HKF equations of state (Helgeson et al., 1981; Tanger and Helgeson, 1988; Shock et al., 1992), the SUPCRT92 software package (Johnson et al., 1992), and thermodynamic data taken from the literature (Shock and Helgeson, 1988, 1990; Shock et al., 1989; Shock, 1995; Amend and Helgeson, 1997; Amend and Plyasunov, 2001; Dick et al., 2006).

## Results

Metabolic heat production rates were measured in crustal fluids by two-step isothermal nanocalorimetry (**Table 2**) as watts of heat flow. The watt unit is defined as the energy consumption rate of one joule per second. Standard deviation of initial and final isothermal measurements provided an estimate of baseline reproducibility of 2.7 nW ml^-1^ and 1.2 nW ml^-1^ in fluids from the JFR flank and NP, respectively. These data were used to determine the detection limit of microbial metabolic heat production rates on crustal fluids and to delimit the minimum temperature (T_min_) and maximum temperature (T_max_) of activity, which were established as 36–85°C in JFR flank fluids and at 34–43°C in NP fluids. The resulting values of Δ*H*_r_ for Reaction (1) were -12.7 to -13.7 kJ (mol SO_4_^2-^)^-1^ from 36 to 85°C, those for Reaction (2) were -444 to -447 kJ (mol O_2_)^-1^ from 34 to 43°C, while those for Reaction (3) were -371 to -373 kJ (mol O_2_)^-1^ from 34 to 43°C.

**Table 2:** Metabolic heat rates measured by two-step isothermal nanocalorimetry.

Crustal fluids	Cardinal temperatures of microbial metabolic heat rates (°C)			Maximum metabolic heat rate^a^	Detection limit	Enthalpy change (Δ*H*_r_)	Cell abundance	Spore-forming cell abundance	Energy turnover^b^
							
	*T*_min_	*T*_max_	*T*_opt_	(nW ml^-1^)	(nW ml^-1^)	(mJ)	(cells ml^-1^)	(cells ml^-1^)	(pW ml^-1^)
Juan de Fuca Ridge (Borehole U1362A)	35.5	85.0	76.2	672	2.7	267.3	8.67*E* + 02	6.66*E* + 04	5.68
North Pond (Borehole U1383C)	34.8	43.0	38.0	285	1.2	12.8	6.88*E* + 05	Not detected	0.18


*^a^Measured at *T*_*opt*_.*

*^b^Calculated from the total metabolic heat generated over the temperature transition time.*

During the transition between initial and final isothermal measurements, metabolic heat production rates increased through a maximum, followed by a continuous decrease (**Figure 2**). Maximum heat production rates of 1.27 μW were determined at a temperature optimum (T_opt_) of 73.5°C in JFR flank fluids, while 0.57 μW were measured at T_opt_ 38°C in NP fluids. Both of these values are ∼15°C above their respective *in situ* temperatures.

**FIGURE 3:**
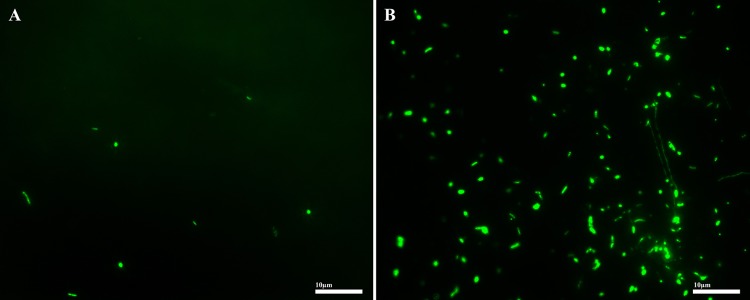
**Epifluorescence-microscopy image of basaltic crustal fluid samples from JFR filtered onto a Whatman 0.2 μm Anodisc filter stained with SYBR Green I, before **(A)** and after **(B)** induction of spore germination by pasteurization and 48 h of incubation at 50°C.** Scale bar 10 μm.

The energy turnover per cell was calculated by dividing the total heat produced during the course of the nanocalorimetry experiment by the number of cells in each fluid sample. Integration of the heat flow temperature plots showed that 267.3 mJ were produced by microbial metabolism in JFR fluids and 12.8 mJ were produced in NP fluids, over 97 and 14 h, respectively. Cellular enumeration within crustal fluids from the JFR flank and NP revealed concentrations of 8.67 × 10^2^ and 6.88 × 10^5^ cells ml^-1^ respectively. However, germination experiments revealed 6.66 × 10^4^ cells ml^-1^ after the pasteurization and incubation of JFR fluids at 50°C (**Figure 3**). Taking into consideration the potential contribution of germinating cells to the measured metabolic heat rates, the cell-specific energy turnover rate in JFR fluids was estimated at 5.68 pW cell^-1^ (**Table 2**). No germinating cells were detected in NP fluids and the cell-specific energy turnover rate was estimated at 0.18 pW cell^-1^ (**Table 2**).

## Discussion

The rate of heat production measured in the nanocalorimeter is equivalent to the heat produced by the sum of all of the reactions occurring in the sample chamber. Essentially, the signal shown in **Figure 2** is the sum of the enthalpies of all of the reactions catalyzed by microorganisms. These heat signals can be combined with computed enthalpies of reactions that are thought to be catalyzed by microorganisms *in situ* to infer rates of metabolic activity. The amount of heat detected in fluids taken from the subseafloor at the JFR flank and at NP differ considerably (**Figure 2**), indicating different underlying processes.

Given that fluids sampled years earlier from the same JFR CORK (Robador et al., 2015) revealed genetic signatures and potential rates of microbial sulfate reduction, it is likely that the heat signals shown in **Figure 2A** are at least partially due to the enthalpy of this process. However, using the range of Δ*H*_r_ values calculated for Reaction (1) and assuming that the total amount of heat measured in the JFR flank sample, 267.3 mJ, was due to sulfate-reduction, the corresponding rates would be 2.4–2.6 × 10^3^ nmol^-1^ ml^-1^_FLUID_ d^-1^, far greater than the gross rates of sulfate reduction previously measured in JFR fluids, ∼0.01 nmol ml^-1^_FLUID_ d^-1^ (Robador et al., 2015). We attribute this large difference in sulfate reduction rates to the germination of thermophilic endospores (6.66 × 10^4^ cells ml^-1^_FLUID_). In fact, thermophilic spore-forming bacteria, including close relatives to members of the sulfate-reducing *Firmicutes* phylum and *Candidatus* Desulforudis lineage, have been reported from these fluids (Robador et al., 2015). Not only can the process of germination potentially add to the heat signal measured, but once these organisms are functional, they can contribute to hydrolytic and fermentative activities which could in turn mediate the production of organic matter and further stimulate the rates of sulfate reduction (Hubert et al., 2009, 2010; de Rezende et al., 2013). Measured heat production rates in this study demonstrate that warm, anaerobic crustal fluids can support a large and active population of these thermophilic bacteria. Given that the entire volume of seawater is estimated to cycle through the ocean crust about every 100,000 years (Johnson and Pruis, 2003), the detection of active marine microorganisms with enhanced survival capacities demonstrates not only the importance of circulating crustal fluids for the passive dispersal on marine microbial biogeography (Müller et al., 2014), but also underscores the physiological potential of cells in crustal fluids to migrate and become established in a new location.

In contrast, the smaller heat signal measured in fluids taken from NP, 12.8 mJ, and narrower thermal range of metabolic activity, 34.8–43.0°C, indicate lower levels of microbial activity than in JFR fluids and a predominately mesophilic community. Although it is noteworthy that a metabolic heat signal was not detected at the *in situ* NP crustal temperature, 3.8°C, metabolic rates at this temperature might be too slow to be measured in the calorimeter. In addition, bacteria are known to be active at temperatures that are greater than those associated with the environment in which they were found by generating sufficient energy from respiration to repair or regenerate temperature-denatured enzymes, ribosomes, etc. (Isaksen and Jørgensen, 1996). Nonetheless, the total heat measured from the NP sample (12.8 mJ) and a median value of Δ*H*_r_ for Reaction (2) show oxygen respiration rates of 24.5 nmol O_2_ ml^-1^_FLUID_ d^-1^. Furthermore, other metabolic processes, i.e., nitrification, which describes the oxidation of NH_4_^+^ to ultimately NO_3_^-^, could also influence oxygen dynamics in NP fluids (Cowen, 2004). Assuming the Δ*H*_r_ values calculated for Reaction (3), the increase in oxygen respiration due to nitrification would yield rates of 29.3 nmol O_2_ ml^-1^_FLUID_ d^-1^. Altogether, these rates validate previous model predictions of microbial activity (0.1 to ∼100 nmol O_2_ ml^-1^_FLUID_ d^-1^, Orcutt et al., 2013b) in the same environmental setting.

It should be noted that when activities of natural populations of bacteria are estimated in terms of sulfate reduction or oxygen consumption, only the energy turnover of respiration is calculated, but there are also energetic costs—and thus heat signals—associated with other cellular processes such as anabolism. In the case of microorganisms in energy-limited environments, anabolic activity is mostly associated with the energy demand for the repair of accumulated damage to key macromolecules, i.e., DNA and housekeeping proteins (Russell and Cook, 1995; Johnson et al., 2007; van Bodegom, 2007), the energetics of which vary considerably depending on the environmental conditions (LaRowe and Amend, 2015c). Differences in cell-specific energy turnover between measured rates in fluids from JFR and NP (5.68 and 0.18 pW cell^-1^, respectively) and compiled cell-specific respiratory rates for anaerobic and aerobic microbes in other deep subsurface environments, ∼3 × 10^-3to^ ^-8^ pW cell^-1^ and ∼1 × 10^-1to^ ^-8^ pW cell^-1^, respectively (Lever et al., 2015), could partly reflect the product of the minimal complement of functions required to sustain a metabolically active state, the basal power requirement (Hoehler and Jørgensen, 2013). Measurements of heat production by direct nanocalorimetry should allow a straightforward interpretation of the basal power requirements of microbes harbored in deeply buried basaltic crustal fluids.

## Author Contributions

AR designed and performed experiments, analyzed data, and wrote the paper. AR performed and analyzed calorimetry data. DL contributed to the analysis of calorimetry data, performed thermodynamic calculations, and contributed to the preparation of the manuscript. SJ, H-TL, and MR were instrumental in the acquisition of fluid samples. AR and JA conceived the study, and JA and KN supported the study. All authors discussed the results and implications and commented on the manuscript at all stages.

## Conflict of Interest Statement

The authors declare that the research was conducted in the absence of any commercial or financial relationships that could be construed as a potential conflict of interest.
